# Comparison of Current Diagnostic Criteria for Acute-On-Chronic Liver Failure

**DOI:** 10.1371/journal.pone.0122158

**Published:** 2015-03-18

**Authors:** Qian Zhang, Ying Li, Tao Han, CaiYun Nie, JunJun Cai, Hua Liu, Ying Liu

**Affiliations:** 1 The Third Central Clinical College of Tianjin Medical University, Tianjin, China; 2 Department of Hepatology, Tianjin Third Central Hospital, Tianjin, China; 3 Tianjin Institute of Hepatobiliary Disease, Tianjin, China; 4 Tianjin Key Laboratory of Artificial Cells, Tianjin, China; Chiba University, Graduate School of Medicine, JAPAN

## Abstract

**Background and Aims:**

Currently, acute-on-chronic liver failure (ACLF) has been defined differently by Asia–Pacific Association for the Study of the Liver (APASL) and Chinese Medical Association (CMA) in the East, as well as EASL-Chronic Liver Failure (EASL-CLIF) Consortium in the West. This study aimed to compare current different diagnostic criteria for ACLF and to determine predictors of the progression into post-enrollment EASL-CLIF ACLF from ACLF at enrollment defined by APASL alone or by both APASL and CMA but not by EASL-CLIF Consortium.

**Methods:**

We retrospectively analyzed clinical data from 394 eligible cirrhotic patients fulfilling at least APASL criteria for ACLF at enrollment. Patient survival was estimated by Kaplan-Meier analysis and subsequently compared by log-rank test. Independent predictors of disease progression were determined using univariate analysis and multivariate Cox regression analysis.

**Results:**

The 90-day mortality rate was 13.1% in patients with ACLF at enrollment defined by APASL alone, 25.3% in patients with ACLF at enrollment defined by both APASL and CMA but not EASL-CLIF Consortium, and 59.3% in patients with ACLF at enrollment defined by EASL-CLIF Consortium in addition to APASL. Baseline Chronic Liver Failure-Sequential Organ Failure Assessment (CLIF-SOFA) score, and the maximum rising rates of CLIF-SOFA score, Model for End-Stage Liver Disease-Sodium (MELD-Na) score and total bilirubin were independent predictors of progression into post-enrollment EASL-CLIF ACLF from ACLF at enrollment defined by APASL alone or by both APASL and CMA but not by EASL-CLIF Consortium.

**Conclusion:**

Different diagnostic criteria for ACLF caused different patient prognosis. So, it is imperative to formulate a unifying diagnostic criteria for ACLF worldwide, thus attaining early identification and treatment, and eventual improvement in survival of ACLF patients. Baseline CLIF-SOFA score, and the maximum rising rates of CLIF-SOFA score, MELD-Na score and total bilirubin may early predict post-enrollment development of EASL-CLIF ACLF.

## Introduction

Acute-on-chronic liver failure (ACLF) is one of the most challenging health problems worldwide, characterized by its rapid progression and dramatically high mortality [1–3]. Unfortunately, current uniform criteria universally accepted for diagnosing ACLF remain unavailable. In the East, there have been two different diagnostic criteria put forward by Asia–Pacific Association for the Study of the Liver (APASL) [4] and Chinese Medical Association (CMA) [5]. In the West, following the European Association for the Study of the Liver and American Association for the Study of Liver Disease (EASL-AASLD) consensus definition [6], the EASL-Chronic Liver Failure (EASL-CLIF) Consortium recently proposed novel diagnostic criteria for ACLF based on a large prospective CANONIC study [2].

Notably, current diagnostic criteria for ACLF differ from each other, creating confusion in the field. Both APASL and CMA define ACLF in terms of acute deterioration of previous chronic liver diseases such as chronic hepatitis and/or cirrhosis [4,5], whereas EASL-CLIF Consortium defines ACLF in terms of predisposed cirrhosis and organ failure(s) associated with worsened liver function [2]. With respect to the two diagnostic criteria in the East, they are also different [4,5]. The APASL criteria for ACLF take a lower cutoff level of serum bilirubin (5 mg/dL) than CMA criteria (10 mg/dL). These differences in diagnostic criteria are not just a matter of semantics but determinants on ACLF identification, timing of treatment and eventual prognosis of ACLF. Hence, it is critically essential to compare APASL, CMA and EASL-CLIF criteria for ACLF, with the consequent potential to gain insight into the future improvement of ACLF prognosis.

Furthermore, in patients with ACLF at enrollment defined by APASL alone or by both APASL and CMA but not by EASL-CLIF Consortium, some patients can recover from the illness following standard medical treatment for ACLF, whereas some patients may progress to ACLF defined by EASL-CLIF Consortium (EASL-CLIF ACLF) after enrollment with worsened clinical and laboratory abnormalities. In this particular group of patients, if impending disease progression can be early predicted and then timely corresponding measures can be adopted, prevention or reversal of this evolutive process would be achieved. Unfortunately, there are no well-established prognostic indicators available for predicting this disease progression thus far.

This present study thus aims to compare APASL, CMA and EASL-CLIF criteria for ACLF, and to determine predictors of the progression into EASL-CLIF ACLF after enrollment in patients with ACLF at enrollment defined by APASL alone or by both APASL and CMA but not by EASL-CLIF Consortium.

## Materials and Methods

### Ethics statement

The study protocol conforms to the ethical guidelines of the Declaration of Helsinki and was approved by Tianjin Third Central Hospital Ethics Committee. Due to the retrospective nature of the study, written informed consent could not be obtained from all patients. All data was de-identified prior to analysis.

### Patients

**Fig 1:**
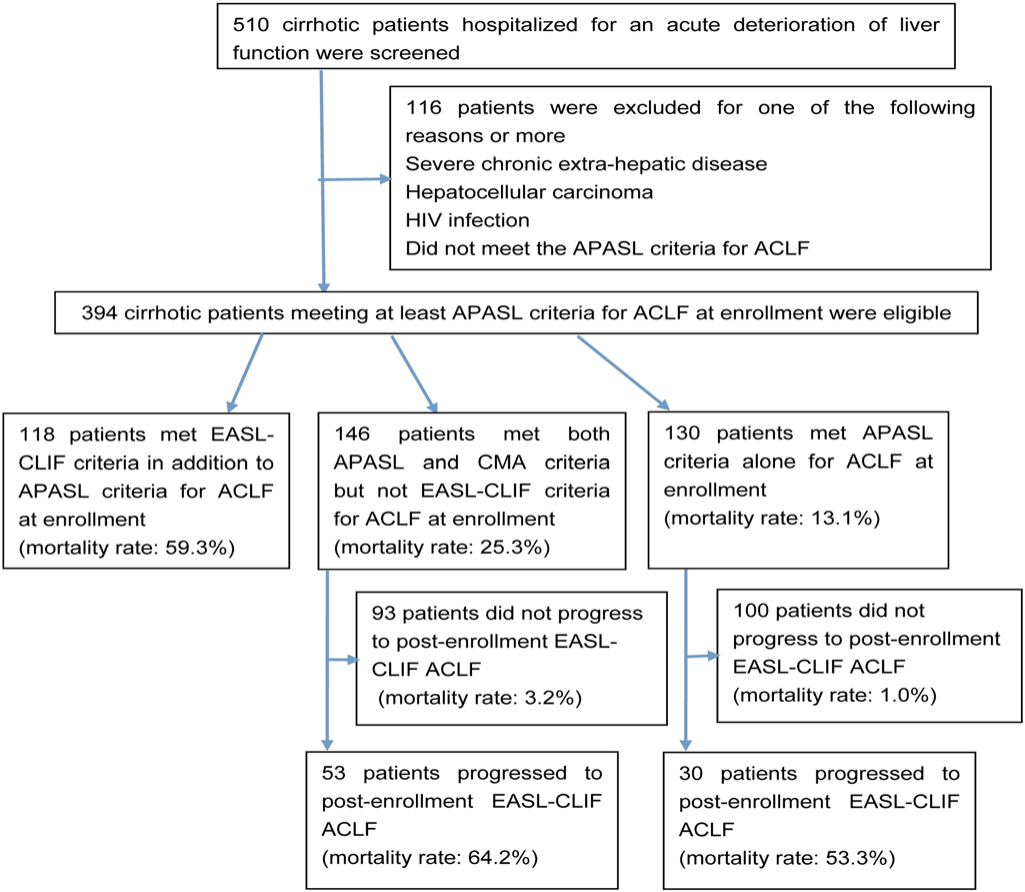
Flowchart of the study group selection process. Abbreviations: ACLF, acute-on-chronic liver failure; APASL, Asia–Pacific Association for the Study of the Liver; CMA, Chinese Medical Association; EASL-CLIF, EASL-Chronic Liver Failure.

The flow chart of the study group selection process is presented in Fig. 1. We retrospectively reviewed data from 510 hospitalized cirrhotic patients with ACLF from January 2008 to May 2014 at Tianjin Third Central Hospital. Of them, 394 cirrhotic patients classified as ACLF at enrollment at least as per APASL criteria were eligible. The remaining 116 patients were excluded because they had severe chronic extra-hepatic disease, hepatocellular carcinoma, HIV infection, or did not meet the APASL criteria for ACLF. The 394 patients received same standard medical interventions, including absolute bed rest, energy supplements and vitamins, intravenous infusion albumin, maintenance water, electrolyte and acid-base equilibrium, prevention and treatment of complications, and so forth. Oral antiviral treatment including Lamivudine, Adefovir Dipivoxil, Telbivudine and Entecavir was administered to the patients in whom hepatitis B virus activated replication. None underwent liver transplantation within 90-day follow-up period.

### Methods

#### Data collection

Overall 394 eligible patients who qualified for at least APASL criteria for ACLF at enrollment were divided into 3 groups: patients satisfying APASL criteria alone for ACLF at enrollment (group A), patients satisfying both APASL and CMA criteria but not EASL-CLIF criteria for ACLF at enrollment (group B), and patients satisfying EASL-CLIF criteria in addition to APASL criteria for ACLF at enrollment (group C). All the patients were followed up from their date of admission until either their death or the end of 90-day follow-up period (Fig. 1).

In all enrolled patients we collected baseline data from demographics, clinical parameters, laboratory values, etiologies of cirrhosis and precipitating events of ACLF at enrollment. In addition, the Child–Turcotte–Pugh (CTP), Model for End-Stage Liver Disease (MELD), MELD-Na and Chronic Liver Failure-Sequential Organ Failure Assessment (CLIF-SOFA) scores were calculated at baseline. After enrollment, in groups A and B patients, post-enrollment precipitating events, clinical parameters, laboratory parameters and severity scores (CTP, MELD, MELD-Na and CLIF-SOFA scores) were routinely collected every 7 days. In addition, the outcome at 90-day follow-up (recovery or death) of each patient was recorded.

#### Procedures

Once data were collected we firstly assessed the prevalence and survival of patients in these 3 groups. Then, as for the patients in groups A and B, we compared the prevalence and survival between patients with and without progression into EASL-CLIF ACLF after enrollment (post-enrollment EASL-CLIF ACLF). Finally, we searched for independent predictive factors associated with the progression into post-enrollment EASL-CLIF ACLF from ACLF at enrollment defined by APASL alone or by both APASL and CMA but not by EASL-CLIF Consortium. We calculated rates of change in clinical indicators (laboratory parameters and severity scores) every 7 days until EASL-CLIF ACLF developed in patients with the progression into post-enrollment EASL-CLIF ACLF, or until MELD score reached to the maximum in patients without this disease progression. Then, we selected the maximum rates of change in each indicator within the study period. For the purpose of this study, baseline clinical characteristics, post-enrollment precipitating events and the maximum rates of change in clinical indicators were compared between patients with and without the progression into post-enrollment EASL-CLIF ACLF.

#### Definitions

The diagnosis of cirrhosis was based on previous liver-biopsy findings or a composite of clinical signs and findings provided by laboratory tests, endoscopy and radiologic imaging.

The APASL criteria for ACLF were defined as [4]: acute hepatic insult manifesting as jaundice (serum bilirubin ≥ 5 mg/dL (85 μmol/L) and coagulopathy (international normalized ratio (INR) ≥ 1.5 or prothrombin activity (PTA) < 40%) complicated within 4 weeks by clinical ascites and/or encephalopathy in a patient with previously diagnosed or undiagnosed chronic liver disease/cirrhosis.

The Chinese criteria for ACLF were defined by CMA as [5]: (1) acute or subacute deterioration of preexisting chronic liver disease; (2) extreme fatigue with severe digestive symptoms (3) progressively worsening jaundice within a short period (serum total bilirubin (TBIL) ≥ 10 mg/dL (171 μmol/L) or a daily elevation ≥ 1 mg/dL (17.1 μmol/L)); (4) an obvious hemorrhagic tendency with PTA ≤ 40% (or INR ≥ 1.50); (5) decompensated ascites; (6) with or without hepatic encephalopathy. According to the CMA staging criteria, patients with ACLF were subdivided into early-stage, intermediate-stage and late-stage ACLF.

Diagnostic criteria and grades of ACLF were defined according to EASL-CLIF Consortium definition, as follows [2]:

No ACLF: (1) patients with no organ failure, or (2) patients with a single “nonkidney” organ failure who had a serum creatinine level < 1.5 mg/dL and no hepatic encephalopathy, or (3) patients with single cerebral failure who had a serum creatinine level < 1.5 mg/dL.

ACLF grade 1: (1) patients with single kidney failure, or (2) patients with single failure of the liver, coagulation, circulation, or respiration who had a serum creatinine level ranging from 1.5 to 1.9 mg/dL and/or mild to moderate hepatic encephalopathy, or (3) patients with single cerebral failure who had a serum creatinine level ranging from 1.5 and 1.9 mg/dL. ACLF grade 2: patients with 2 organ failures.

ACLF grade 3: patients with 3 organ failures or more.

Organ failure was defined based on the CLIF-SOFA score [2].

The CLIF-SOFA score was created by EASL-CLIF Consortium and included subscores ranging from 0 to 4 for each of six components (liver, kidneys, brain, coagulation, circulation and lungs) [2].

The CTP score of patients was calculated by rating the following parameters from 1 to 3: ascites, encephalopathy, prothrombin time (< 15, 15–17, > 17 s), serum bilirubin (< 34, 34–51, > 51 μmol/L), and serum albumin (> 35, 28–35, < 28 g/L) [7].

The MELD score was calculated according to the Malinchoc formula: MELD score = 3.78 × log_e_ (bilirubin [mg/dL]) + 11.2 × log_e_ (INR) + 9.57 × log_e_ (creatinine [mg/dL]) + 6.43 × (etiology: 0 if cholestatic or alcoholic, 1 otherwise) [8].

The MELD-Na score was calculated according to the following formula: MELD-Na = MELD + 1.59 × (135—serum sodium), where the serum sodium concentration is bound between 125 and 135 mmol per liter [9].

#### Statistical analyses

Categorical variables were expressed as number (%), and continuous variables were described as mean ± SD or median (inter-quartile range, Q1-Q3).

Patient survival was estimated by Kaplan-Meier analysis and subsequently was compared by log-rank test.

We performed univariate analysis (using Chi-squared test or the Fisher exact test for categorical variables, Mann–Whitney U tests for not normal continuous parameters and Student’ s t test for normal continuous parameters) to compare characteristics of patients with and without progression to post-enrollment EASL-CLIF ACLF. The variables that were found to be statistically different between them were included as candidate variables in a forward conditional multivariate Cox regression analysis to investigate independent predictors of disease progression. For this analysis, the conditional probabilities for stepwise entry and removal of a factor were 0.05 and 0.10, respectively.

All the statistical analyses were performed with SPSS 20.0 (SPSS, Chicago, IL, USA) software. For all analyses, P value < 0.05 was considered to be statistically significant.

## Results

### Patients with ACLF in the whole series

A total of 510 hospitalized cirrhotic patients were screened of whom 394 were eligible. Table 1 exhibits characteristics at enrollment of the overall study populations. The mean age was 49.5 ± 11.0 years, and the patients were predominantly men (76.1%). The most common etiology of cirrhosis was hepatitis B (52.5%), followed by alcoholic (37.1%). The most frequent precipitating event of ACLF was bacterial infection (58.4%), followed by superimposed viral hepatitis or reactivation of hepatitis (33.5%) and alcohol (23.4%).

**Table 1:** Characteristics of eligible patients at enrollment.

Characteristics	
**Age (years)**	49.5±11.0
**Male**	300(76.1%)
**Etiology of cirrhosis**	
Hepatitis B	207(52.5%)
Hepatitis C	27(6.9%)
Alcoholic	146(37.1%)
Alcoholic plus hepatitis B	26(6.6%)
Autoimmune liver disease	32(8.1%)
Cryptogenic	10(2.5%)
**Potential precipitating events**	
Bacterial infection	230(58.4%)
Superimposed viral hepatitis or reactivation of hepatitis	132(33.5%)
Alcohol	92(23.4%)
Gastrointestinal hemorrhage	54(13.7%)
Hepatotoxic drugs	21(5.3%)
**Clinical parameters**	
Ascites	221(56.1%)
HE	67(17.0%)
Electrolytedisturbances	292(74.1%)
**Type of organ failure** ^†^	
Liver	189(48.0%)
Coagulation	130(33.0%)
Cerebral	38(9.6%)
Kidney	28(7.1%)
Circulation	6(1.5%)
Lungs	4(1.0%)
**Laboratory parameters**	
WBC (×10^9^/L)	6.5(4.4–9.9)
PLT (×10^9^/L)	74.0(47.8–108.8)
ALB (g/L)	27.8±4.9
ALT (U/L)	66.0(35.0–255.5)
TBIL (μmol/L)	198.1(131.3–306.4)
BUN (mmol/L)	5.6(3.8–9.0)
Cr (μmol/L)	60.0(49.0–82.3)
Serum sodium (mmol/L)	134.0(129.8–137.1)
INR	2.2(1.8–2.6)
PT (sec)	23.8(20.6–27.6)
**Severity scores**	
CTP	12.0(11.0–13.0)
MELD	19.0(14.0–23.0)
MELD-Na	20.9(16.0–28.3)
CLIF-SOFA	7.0(6.0–8.0)

Categorical variables expressed as number (%), non-normal continuous variables as median (Q1–Q3) and normal continuous variables as mean ± SD.

HE, hepatic encephalopathy; HRS, hepatorenal syndrome; WBC, white blood cells; PLT, platelet; ALB, albumin; ALT, alanine aminotransferase; TBIL, total bilirubin; BUN, blood urea nitrogen; Cr, creatine; INR, international normalized ratio; PT, prothrombin time; CTP, child-turcotte-pugh; MELD, model for end-stage liver disease; CLIF-SOFA, chronic liver failure-sequential organ failure assessment.

^†^Organ failure was defined based on the CLIF-SOFA score.

### Prevalence and survival associated with ACLF at enrollment

At enrollment, among 394 eligible patients, there were 130 patients (33.0%) in group A, 146 patients (37.1%) in group B, and 118 patients (29.9%) in group C.

**Fig 2:**
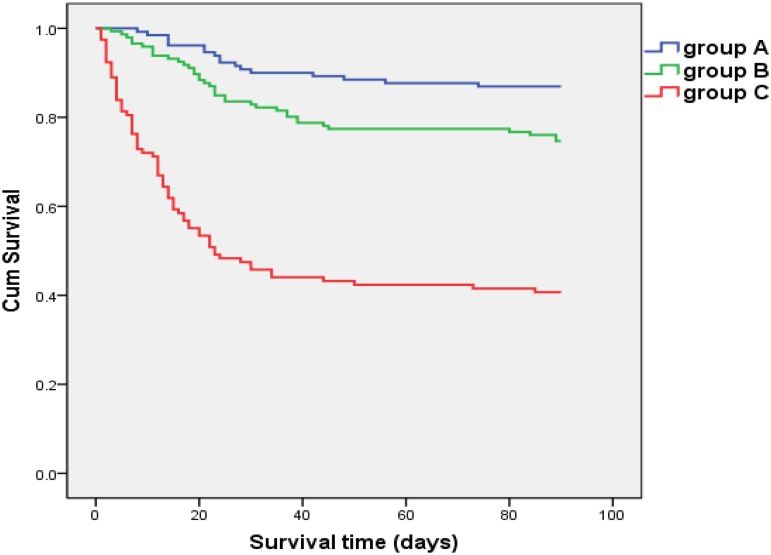
Comparison of survival among patients with ACLF at enrollment defined by different criteria. Overall 394 eligible patients who qualified for at least APASL criteria for ACLF at enrollment were divided into 3 groups: patients satisfying APASL criteria alone for ACLF at enrollment (group A), patients satisfying both APASL and CMA criteria but not EASL-CLIF criteria for ACLF at enrollment (group B), and patients satisfying EASL-CLIF criteria in addition to APASL criteria for ACLF at enrollment (group C). In comparison with patients in group A and group B, the 90-day survival was significantly lower for patients in group C (log-rank test: P < 0.001). Besides, significantly lower survival was also observed for patients in group B, as compared to patients in group A (log-rank test: P < 0.05).

The 90-day mortality rate was 13.1%, 25.3% and 59.3% in group A, group B and group C, respectively (Fig. 1). Compared to patients in group A and group B, those in group C were observed to have a significantly lowered survival (log-rank test: P < 0.001). Besides, the survival of patients in group B was significantly lower than that of patients in group A (log-rank test: P < 0.05) (Fig. 2). The median survival time of patients in group A, group B and group C were >90 days, >90 days and 23 days, respectively.

### Post-enrollment EASL-CLIF ACLF

Out of the entire 276 patients in groups A and B, 83 (30.1%) progressed to post-enrollment EASL-CLIF ACLF (Fig. 1).

**Fig 3:**
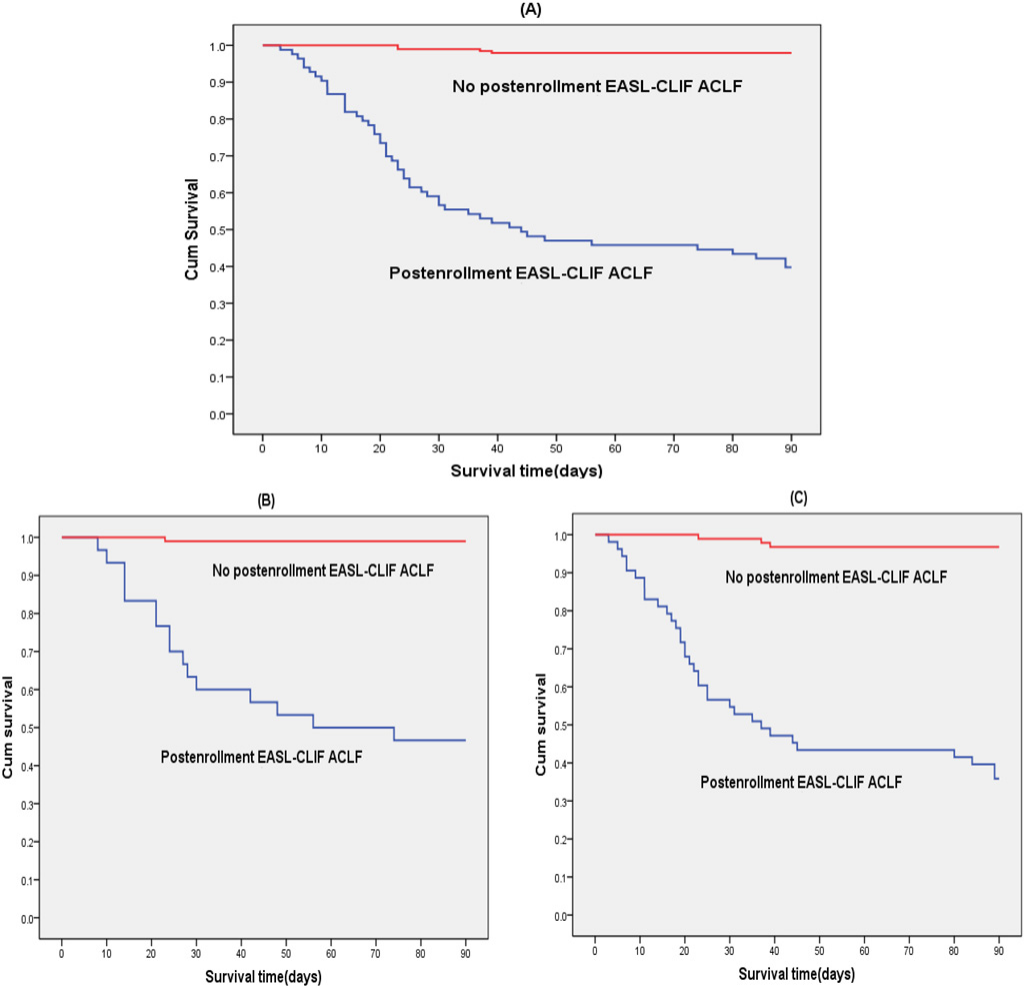
Comparison of survival between patients with and without progression to post-enrollment EASL-CLIF ACLF. Patients with ACLF at enrollment defined by APASL criteria alone were classified into group A, while patients with ACLF at enrollment defined by both APASL and CMA criteria but not EASL-CLIF criteria were classified into group B. Among the entire 276 patients in groups A and B, patients with progression to post-enrollment EASL-CLIF ACLF had a significantly lower survival than those without (log-rank test: P < 0.001) (A). Among patients in either group A (B) or group B (C), significantly lower survival was also observed in patients with progression to post-enrollment EASL-CLIF ACLF than those without (log-rank test: P < 0.001). Abbreviations: ACLF, acute-on-chronic liver failure; EASL-CLIF, EASL-Chronic Liver Failure.

As for overall 276 patients in groups A and B, 90-day mortality rates of patients who did and did not progress to post-enrollment EASL-CLIF ACLF were 60.2% and 2.1%, respectively. The Kaplan-Meier analysis showed that, patients with progression to post-enrollment EASL-CLIF ACLF had a significant shorter median survival time than those without: 44 days vs. over 90 days (log-rank test: P < 0.001). Similarly, in either group A or group B, 90-day survival was also significantly worsened in patients with progression to post-enrollment EASL-CLIF ACLF compared to those without (log-rank test: P < 0.001). The median survival time of patients with and without progression to post-enrollment EASL-CLIF ACLF were 56 days vs. over 90 days in group A, and 37 days vs. over 90 days in group B, respectively. The 90-day survival curves of patients with and without progression to post-enrollment EASL-CLIF ACLF are provided in Fig. 3.

### Analysis of the whole group of patients with EASL-CLIF ACLF

**Fig 4:**
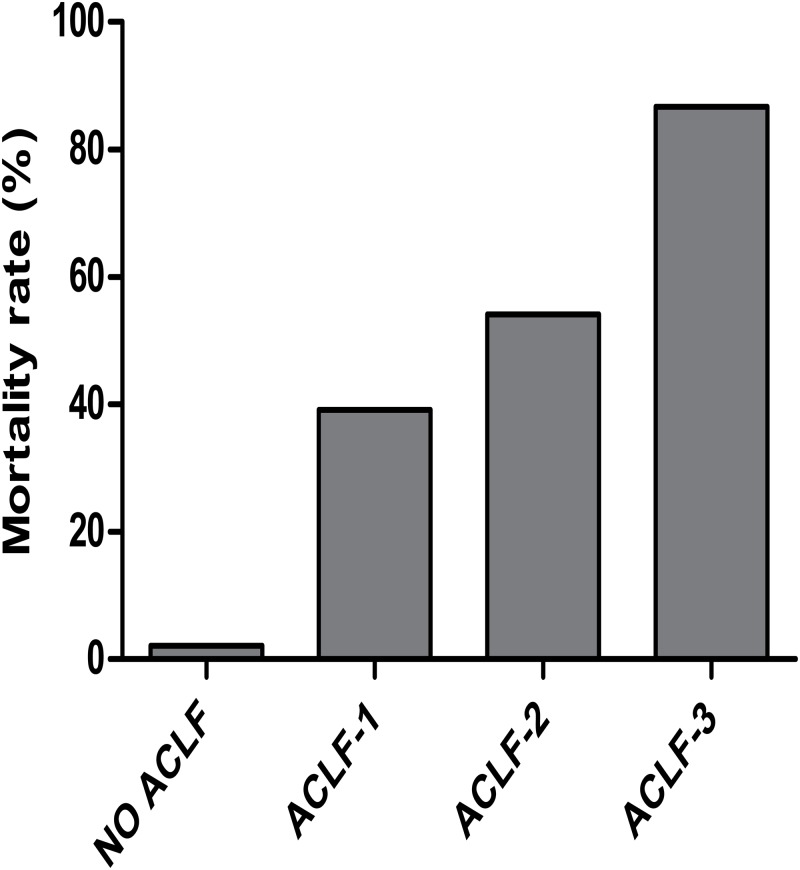
Mortality rate at 90 days according to the grade of ACLF defined by EASL-CLIF Consortium. Among patients identified as EASL-CLIF ACLF either at enrollment or after enrollment, the 90-day mortality rate was 39.1% for grade 1, 54.1% for grade 2, 86.7% for grade 3, respectively. The 90-day mortality rate in patients without EASL-CLIF ACLF both at enrollment and after enrollment was 2.1%**.** Abbreviations: ACLF, acute-on-chronic liver failure.

A total of 201 patients (51.0%) had EASL-CLIF ACLF either at enrollment or after enrollment (Fig. 1); 23 (11.4%) were defined as having ACLF grade 1, 133 (66.2%) as grade 2, and 45 (22.4%) as grade 3. Fig. 4 shows that 90-day mortality rate in patients with ACLF was 59.7% (39.1% for grade 1, 54.1% for grade 2, 86.7% for grade 3). The 90-day mortality rate in patients without ACLF at enrollment or after enrollment was 2.1%.

### Factors associated with progression into post-enrollment EASL-CLIF ACLF in patients with ACLF at enrollment defined by APASL alone or by both APASL and CMA but not by EASL-CLIF Consortium: univariate analysis

Univariate analysis showed that eight baseline characteristics differed statistically between the patients with and without progression into post-enrollment EASL-CLIF ACLF from ACLF at enrollment defined by APASL alone or by both APASL and CMA but not by EASL-CLIF Consortium. These included baseline WBC, ALT, INR, PT, MELD, MELD-Na, CLIF-SOFA and bacterial infection at enrollment (Table 2). Furthermore, post-enrollment bacterial infection, superimposed viral hepatitis or reactivation of hepatitis, gastrointestinal hemorrhage, as well as the maximum rates of change in PLT, TBIL, INR, PT, CTP, MELD, MELD-Na and CLIF-SOFA were also significantly different between them (Table 3).

**Table 2:** Comparison of baseline characteristics between patients with and without progression to post-enrollment EASL-CLIF ACLF.

Characteristics at baseline	Patients with progression to post-enrollment EASL-CLIF ACLF (N = 83)	Patients without progression to post-enrollment EASL-CLIF ACLF (N = 193)	P value^†^
**Age(years)**	49.4±9.9	49.7±10.8	0.81
**Male sex**	63(75.9%)	142(71.7%)	0.69
**Etiology of cirrhosis**			
Hepatitis B	44(53.0%)	102(52.8%)	0.98
Hepatitis C	8(9.6%)	12(6.2%)	0.32
Alcoholic	29(28.2%)	69(35.8%)	0.19
Alcoholic plus hepatitis B	8(9.6%)	14(7.3%)	0.50
Autoimmune liver disease	5(6.0%)	21(10.9%)	0.21
Cryptogenic	6(7.2%)	4(2.1%)	0.08
**Precipitating events**			
Bacterial infection	50(60.2%)	89(46.1%)	0.03
Superimposed viral hepatitis or reactivation of hepatitis	36(43.4%)	74(38.3%)	0.43
Alcohol	18(21.7%)	49(25.4%)	0.51
Gastrointestinal hemorrhage	12(14.5%)	15(7.8%)	0.09
Hepatotoxic drugs	6(7.2%)	11(5.7%)	0.63
**Clinical parameters**			
Ascites	64(77.1%)	145(75.1%)	0.73
HE	5(6.0%)	12(6.2%)	0.95
Electrolyte disturbances	65(78.3%)	129(66.8%)	0.06
**Laboratory parameters**			
WBC (×10^9^/L)	6.5(5.0–8.9)	5.8(3.9–8.4)	0.03
PLT (×10^9^/L)	78.0(47.0–117.0)	77.0(52.0–118.0)	0.78
ALB (g/L)	28.9±4.0	27.9±4.8	0.09
ALT (U/L)	84.0(37.0–485.0)	56.0(32.0–250.0)	0.03
TBIL (μmol/L)	183.3(131.8–305.5)	162.6(120.8–252.0)	0.06
BUN (mmol/L)	5.3(3.8–8.2)	5.0(3.6–7.0)	0.26
Cr (μmol/L)	58.0(49.0–73.0)	56.0(48.0–72.0)	0.53
Serum sodium (mmol/L)	134.2(130.2–136.2)	135.0(131.3–137.6)	0.10
INR	2.1(1.8–2.3)	1.9(1.7–2.3)	0.04
PT (sec)	22.9(20.7–25.3)	21.9(19.4–24.7)	0.03
**Severity scores**			
CTP	12(10–12)	11(10–12)	0.53
MELD	17.8±6.0	16.0±5.3	0.02
MELD-Na	20.1(17.8–27.6)	18.7(14.0–23.8)	0.02
CLIF-SOFA	7(6–7)	7(6–7)	0.01

Categorical variables expressed as number (%), non-normal continuous variables as median (Q1–Q3) and normal continuous variables as mean ± SD.

ACLF, acute-on-chronic liver failure; HE, hepatic encephalopathy; HRS, hepatorenal syndrome; WBC, white blood cells; PLT, platelet; ALB, albumin; ALT, alanine aminotransferase; TBIL, total bilirubin; BUN, blood urea nitrogen; Cr, creatine; INR, international normalized ratio; PT, prothrombin time; CTP, child-turcotte-pugh; MELD, model for end-stage liver disease; CLIF-SOFA, chronic liver failure-sequential organ failure assessment.

^†^P value of comparisons between patients with and without progression to post-enrollment EASL-CLIF ACLF.

**Table 3:** Comparison of characteristics after enrollment between patients with and without progression to post-enrollment EASL-CLIF ACLF.

Characteristics	Patients with progression to post-enrollment EASL-CLIF ACLF (N = 83)	Patients without progression to post-enrollment EASL-CLIF ACLF (N = 193)	P value^†^
**Post-enrollment precipitating events**			
Bacterial infection	66(79.5%)	69(35.8%)	<0.001
Superimposed viral hepatitis or reactivation of hepatitis	33(39.8%)	53(27.5%)	0.04
Alcohol	17(20.5%)	33(17.1%)	0.50
Gastrointestinal hemorrhage	17(20.5%)	10(5.2%)	<0.001
Hepatotoxic drugs	5(6.0%)	7(3.6%)	0.37
**Maximum rate of change in laboratory parameters**			
V_m_ (ΔWBC) [×10^9^/(L·d)]	0.05(-0.06–0.25)	0.01(-0.13–0.20)	0.14
V_m_ (ΔPLT) [×10^9^/(L·d)]	-1.00(-3.14–0.08)	-0.25(-2.24–1.05)	0.02
V_m_ (ΔALB) [g/(L·d)]	0.14(-0.12–0.41)	0.20(-0.05–0.45)	0.25
V_m_ (ΔALT) [U/(L·d)]	-1.98(-15.17–0.45)	-0.83(-9.77–0.14)	0.51
V_m_ (ΔTBIL) [μmol/(L·d)]	4.99(1.40–12.80)	-0.06(-3.17–3.27)	<0.001
V_m_ (ΔINR) (1/d)	0.04(0.01–0.12)	0.00(-0.03–0.02)	<0.001
V_m_ (ΔPT) (sec/d)	0.26(0.03–0.88)	0.00(-0.27–0.16)	<0.001
V_m_ (ΔBUN) [μmol/(L·d)]	0.14(-0.05–0.46)	0.07(-0.08–0.25)	0.06
V_m_ (ΔCr) [μmol/(L·d)]	1.00(-0.44–4.23)	0.57(-0.33–2.13)	0.16
V_m_ (ΔSerum sodium) [mmol/(L·d)]	**-**0.05(-0.36–0.21)	0.08(-0.28–0.38)	0.07
**Maximum rate of change in severity scores**			
V_m_ (ΔCTP) (1/d)	0.01(0.00–0.17)	0.00(-0.14–0.00)	<0.001
V_m_ (ΔMELD) (1/d)	0.57(0.06–1.03)	0.08(-0.21–0.30)	<0.001
V_m_ (ΔMELD-Na) (1/d)	0.58(0.06–1.49)	-0.01(-0.38–0.40)	<0.001
V_m_ (ΔCLIF-SOFA) (1/d)	0.19(0.09–0.43)	0.00(-0.03–0.00)	<0.001

Categorical variables expressed as number (%), non-normal continuous variables as median (Q1–Q3) and normal continuous variables as mean ± SD.

ACLF, acute-on-chronic liver failure; WBC, white blood cells; PLT, platelet; ALB, albumin; ALT, alanine aminotransferase; TBIL, total bilirubin; BUN, blood urea nitrogen; Cr, creatine; INR, international normalized ratio; PT, prothrombin time; CTP, child-turcotte-pugh; MELD, model for end-stage liver disease; CLIF-SOFA, chronic liver failure-sequential organ failure assessment.

^†^P value of comparisons between patients with and without progression to post-enrollment EASL-CLIF ACLF.

V_m_ (Δ indicator) was used to represent the maximum rate of change in clinical indicators.

### Factors associated with progression into post-enrollment EASL-CLIF ACLF in patients with ACLF at enrollment defined by APASL alone or by both APASL and CMA but not by EASL-CLIF Consortium: multivariate Cox regression analysis

As shown in Table 4, baseline CLIF-SOFA score, and the maximum rising rates of CLIF-SOFA score, MELD-Na score and TBIL level were independent predictors of progression into post-enrollment EASL-CLIF ACLF from ACLF at enrollment defined by APASL alone or by both APASL and CMA but not by EASL-CLIF Consortium.

**Table 4:** Multivariate Cox regression analysis of independent predictors associated with progression into post-enrollment EASL-CLIF ACLF from ACLF at enrollment defined by APASL alone or by both APASL and CMA but not by EASL-CLIF Consortium.

Variables	HR	95% CI	P value
Baseline CLIF-SOFA	1.326	1.044–1.685	0.021
V_m_ (ΔCLIF-SOFA)	13.419	7.319–25.221	<0.001
V_m_ (ΔMELD-Na)	1.047	1.021–1.074	<0.001
V_m_ (ΔTBIL)	1.343	1.161–1.553	<0.001

ACLF, acute-on-chronic liver failure; HR, hazard ratio; CI, confidence interval; V_m_ (Δ indicator) was used to represent the maximum rate of change in clinical indicators; MELD, model for end-stage liver disease; TBIL, total bilirubin; CLIF-SOFA, chronic liver failure-sequential organ failure assessment.

## Discussion

ACLF carries an extraordinarily high mortality, but its precise diagnostic criteria remain ambiguous. Currently, the differences in diagnostic criteria for ACLF have hampered international academic exchange and comparability among studies, thereby leading to considerable regional discrepancies in ACLF identification, onset treatment timing and eventual prognosis. Therefore, there is an urgent need to make a comparison in different diagnostic criteria for ACLF (APASL, CMA and EASL-CLIF criteria), with the hope to develop a homogeneous diagnostic criteria worldwide and then to attain future survival improvement of ACLF.

The differences in East-West diagnostic criteria for ACLF largely reflect the regional variation in underlying chronic liver disease and acute insults of ACLF [1,3–5]. As indicated from the CANONIC study, in the West, alcoholic cirrhosis is regarded as the commonest underlying chronic liver diseases, and the precipitants of ACLF are mostly bacterial infection and alcohol [2]. However, as showed from the data of APASL ACLF Research Consortium (AARC), in the East, the majority of ACLF is precipitated by hepatitis B reactivation and super-infection with Hepatitis E virus, superimposed on underlying chronic liver disease, which is not necessarily cirrhosis [4]. In our Asian ACLF cohort of this study, bacterial infection constituted the most predominant precipitating events of ACLF rather than reactivation of hepatitis B or superimposed viral hepatitis. A plausible explanation could be that since EASL-CLIF criteria for ACLF emphasizes predisposed cirrhosis although both APASL and CMA criteria do not [2,4,5], only cirrhotic patients were enrolled in this study in order to unify chronic liver disease background at enrollment of the study patients, and it is well-known that cirrhotic patients are more likely to develop infection than the general population [4,10].

Furthermore, the results of our present study clearly validated that, the probability of 90-day mortality was high in patients with ACLF at enrollment defined by EASL-CLIF Consortium in addition to APASL, intermediate in patients with ACLF at enrollment defined by both APASL and CMA but not by EASL-CLIF Consortium, and very low in patients with ACLF at enrollment defined by APASL alone. The markedly different prognosis among these ACLF patients may be mostly attributed to the considerable heterogeneity in the severity of ACLF at enrollment diagnosed by different criteria. According to the Eastern criteria for ACLF (proposed by either APASL or CMA), liver failure alone is focused on, and the lower cutoff levels of INR (i.e., >1.5) and serum bilirubin (i.e., 5 mg/dL defined by APASL or 10 mg/dL defined by CMA) are taken to define liver failure [4,5]. However, according to the Western criteria (proposed by EASL-CLIF Consortium), the occurrence of multi-system organ dysfunctions and failures is underlined, and the thresholds for the diagnosis of organ failure are very stringent based on CLIF-SOFA score (i.e., liver failure defined as bilirubin ≥12 mg/dL), resulting in increased mortality [2]. Additionally, quite a number of studies have indicated that the greater the number of organ dysfunction or failure at diagnosis, the lower the ACLF patient survival [2–4,11–16]. As with the observations of CANONIC study [2], this study also confirmed a clear trend for an increase in 90-day mortality in parallel to the increase in EASL-CLIF ACLF grade. Together these results suggest that prognosis of ACLF was closely related to the stage at which the disease is detected and managed, as early intervention can timely reduce or correct hepatic injury and contribute to a significant improvement in prognosis [1,5,15,17,18]. However, the existing variability in the definition of ACLF poses a considerable obstacle to early identification and treatment of ACLF patients. Thus, future endeavors should be targeted at formulating a worldwide consensus definition of ACLF.

Another intriguing and important finding of this study was significantly different disease progression among patients with ACLF at enrollment defined by APASL alone or by both APASL and CMA but not by EASL-CLIF Consortium, despite receiving same standard medical treatment for ACLF. Of them, some patients recovered to the state they was in prior to the acute insult, but unfortunately, some patients aggravated to more severe state with worsened clinical and laboratory indicators and eventually progressed to post-enrollment EASL-CLIF ACLF with multi-system organ dysfunctions and failures. Our data clearly demonstrated a marked reduction in survival of patients with disease progression compared to no progression. Based on the powerful association between the post-enrollment EASL-CLIF ACLF development and worsened prognosis, we explored risk factors predictive of this progression to achieve early recognition and timely intervention, thereby preventing or reversing this progression and ultimately attaining improvement in survival. As a result, four risk factors were found independently predictive, including baseline CLIF-SOFA score, and the maximum rising rates of CLIF-SOFA score, MELD-Na score and TBIL level.

The CLIF-SOFA score, which is created through adapting definitions of the original SOFA subscores to patients with cirrhosis, has been widely used to assess the number of organ dysfunctions and failures [2]. Higher scores indicated more severe organ impairment and predicted more likelihood of disease progression. This study documented that both baseline CLIF-SOFA score and the maximum rising rate of CLIF-SOFA score were significantly related to post-enrollment EASL-CLIF ACLF development from ACLF at enrollment defined by APASL alone or by both APASL and CMA but not by EASL-CLIF Consortium.

The MELD score, which was initially formulated to assess the short-term prognosis of cirrhotic patients undergoing the transjugular intrahepatic portosystemic shunt [8], is now widely accepted as a high-potency prognostic scoring system for assessing short-term mortality in a broad spectrum of liver diseases [19–21], and its dramatic increase over time has also been demonstrated the ability to predict a poor outcome in ACLF patients [22–24]. However, the use of MELD score alone would underestimate the illness severity and mortality of ACLF patients with hemodynamic derangement, because the MELD score does not contain the assessment of abnormal hemodynamic states which are commonly found in ACLF patients [25,26]. Thus, MELD-Na, incorporating serum sodium concentration, which indirectly reflects the hemodynamic status, was created to remedy this shortcoming. Numerous studies have also provided clear evidence that MELD-Na score can improve the prediction of short, medium, and long term mortality in cirrhotic patients over the traditional MELD score [27–32]. The variation in MELD-Na values over time can correspond to dynamic changes in liver function, and in the present study, its maximum rising rate was identified as a decisive indicator for predicting the disease progression into post-enrollment EASL-CLIF ACLF, thereby allowing more aggressive therapy.

Additionally, the findings of the present study confirmed the prognostic value of the maximum rising rate of TBIL level in unfavorable evolution into post-enrollment EASL-CLIF ACLF. As is well known, the dramatic jaundice increase has an extremely intimate relationship with a decreased hepatic detoxification function [3,4,15,17]. Besides jaundice, another hallmark of acute exacerbation in liver function is coagulopathy, which results from impaired synthesis and increased consumption of coagulation factors [3,4,15,17]. In this study, the PT displayed significantly accelerated growth in patients with disease progression compared to patients without in univariate analysis. However, in multivariate analysis, it was not an independent predictor, which may be related to the relatively small sample size.

In conclusion, although this study has its limitations by its retrospective nature, it enables differences of ACLF defined by APASL, CMA and EASL-CLIF Consortium to be clarified, and reveals that the variability in the ACLF definition eventually results in diverse prognosis of ACLF. Therefore, considerable efforts are urgently needed to develop a single uniform definition of ACLF worldwide that would facilitate international research and academic exchange, and allow consensus regarding diagnosis and optimized treatment to be built, eventually contributing to improved outcomes of ACLF. Moreover, this study demonstrates that baseline CLIF-SOFA score, and the maximum rising rates of CLIF-SOFA score, MELD-Na score and TBIL level can effectively provide early predictive information on the disease progression into post-enrollment EASL-CLIF ACLF from ACLF at enrollment defined by APASL alone or by both APASL and CMA but not by EASL-CLIF Consortium, thereby allowing physicians to escalate treatment in a timely manner to prevent deleterious disease progression and improve patient prognosis.
