# Correction: Comparison of Current Diagnostic Criteria for Acute-On-Chronic Liver Failure

**DOI:** 10.1371/journal.pone.0133817

**Published:** 2015-07-20

**Authors:** Qian Zhang, Ying Li, Tao Han, CaiYun Nie, JunJun Cai, Hua Liu, Ying Liu

The affiliation for the second author is incorrect. Ying Li is also affiliated with #1 The Third Central Clinical College of Tianjin Medical University, Tianjin, China in addition to affiliations #2, #3, and #4.
